# Interplay between mTOR and Purine Metabolism Enzymes and Its Relevant Role in Cancer

**DOI:** 10.3390/ijms25126735

**Published:** 2024-06-19

**Authors:** Simone Allegrini, Marcella Camici, Mercedes Garcia-Gil, Rossana Pesi, Maria Grazia Tozzi

**Affiliations:** 1 Unità di Biochimica, Dipartimento di Biologia, Università di Pisa, Via San Zeno 51, 56127 Pisa, Italy; marcella.camici@unipi.it (M.C.); rossana.pesi@unipi.it (R.P.); maria.grazia.tozzi@unipi.it (M.G.T.); 2 Centro di Ricerca Interdipartimentale Nutrafood “Nutraceuticals and Food for Health”, Università di Pisa, 56126 Pisa, Italy; mercedes.garcia@unipi.it; 3 CISUP, Centro per l’Integrazione Della Strumentazione Dell’Università di Pisa, 56127 Pisa, Italy; 4 Unità di Fisiologia Generale, Dipartimento di Biologia, Università di Pisa, Via San Zeno 31, 56127 Pisa, Italy

**Keywords:** mTOR, purine metabolism, cancer, purinosome, one-carbon metabolism, mitochondria, c-Myc, AKT, cell survival, proliferation

## Abstract

Tumor cells reprogram their metabolism to meet the increased demand for nucleotides and other molecules necessary for growth and proliferation. In fact, cancer cells are characterized by an increased “de novo” synthesis of purine nucleotides. Therefore, it is not surprising that specific enzymes of purine metabolism are the targets of drugs as antineoplastic agents, and a better knowledge of the mechanisms underlying their regulation would be of great help in finding new therapeutic approaches. The mammalian target of the rapamycin (mTOR) signaling pathway, which is often activated in cancer cells, promotes anabolic processes and is a major regulator of cell growth and division. Among the numerous effects exerted by mTOR, noteworthy is its empowerment of the “de novo” synthesis of nucleotides, accomplished by supporting the formation of purinosomes, and by increasing the availability of necessary precursors, such as one-carbon formyl group, bicarbonate and 5-phosphoribosyl-1-pyrophosphate. In this review, we highlight the connection between purine and mitochondrial metabolism, and the bidirectional relation between mTOR signaling and purine synthesis pathways.

## 1. Introduction

**Figure 1:**
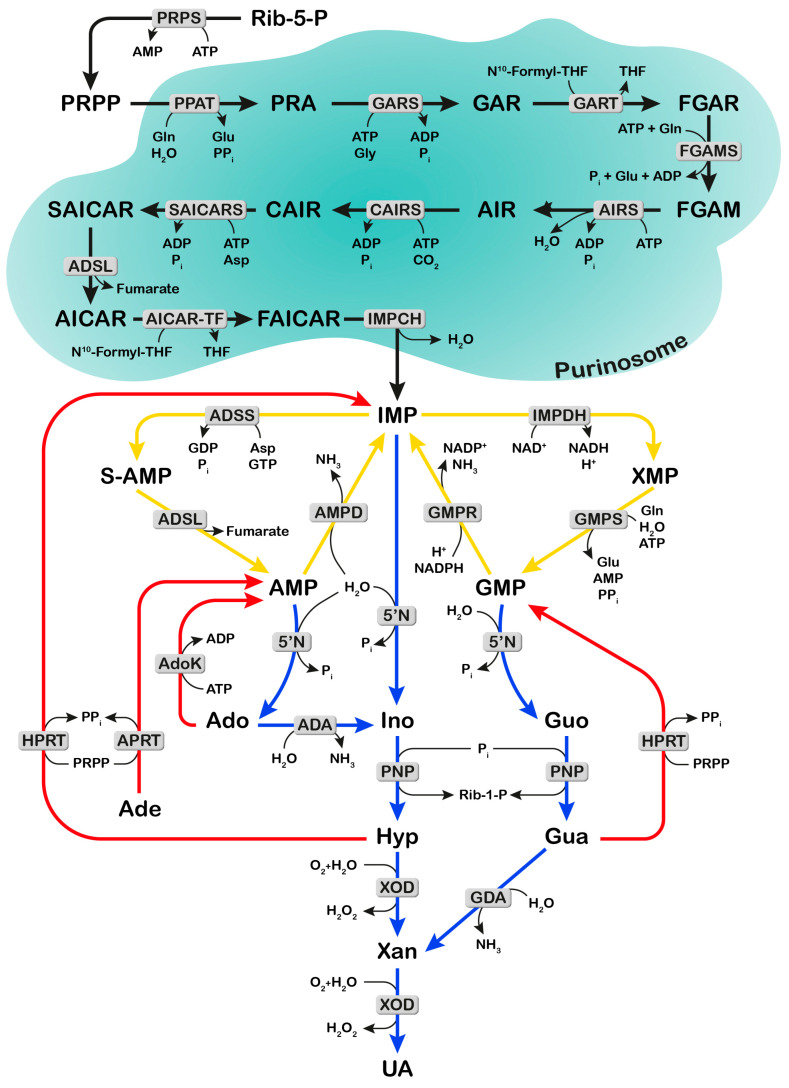
Purine synthesis, interconversion and degradation pathways. Black arrows: “de novo” synthesis. PRPP (5-phosphoribosyl-1-pyrophosphate) is synthesized from Rib-5-P (ribose-5-phosphate) by PRPS (PRPP synthetase). Ten steps are required to convert PRPP into IMP: PPAT (PRPP amidotransferase), GARS (glycinamide ribonucleotide synthetase) GART (GAR transformylase), FGAMS (formylglycinamidine ribonucleotide synthetase), AIRS (aminoimidazole ribonucleotide synthetase), CAIRS (carboxyAIRS), SAICARS (succinyl aminoimidazole carboxamide ribonucleotide synthetase), ADSL (adenylosuccinate lyase), AICAR-TF (AICAR formyltransferase) and IMPCH (IMP cyclohydrolase). Gln: glutamine; Glu: glutamate; gly: glycine; THF: tetrahydrofolate. Yellow arrows: interconversion pathways. “AMP cycle”: ADSS (adenylosuccinate synthetase), ADSL, AMPD (AMP deaminase). “GMP cycle”: IMPDH (IMP dehydrogenase), GMPS (GMP synthetase), GMPR (GMP reductase). Asp: Aspartate. Blue arrows: degradation pathways. 5′N (cytosolic 5′-nucleotidase) converts IMP, AMP and GMP into their respective nucleosides: Ino (inosine), Ado (adenosine) and Guo (guanosine). PNP (purine nucleoside phosphorylase) converts Ino and Guo into their bases Hyp (hypoxanthine) and Gua (guanine). XOD (xanthine oxidase) converts Hyp into Xan (xanthine) and UA (uric acid). ADA: adenosine deaminase; GDA: guanine deaminase; Rib-1-P: ribose-1-phosphate. Red arrows: salvage pathways. HPRT (hypoxanthine-guanine phosphoribosyltransferase) converts Gua and Hyp into GMP and IMP. APRT (adenine phosphoribosyltransferase) converts Ade (adenine) into AMP, and AdoK (adenosine kinase) converts Ado into AMP.

**Figure 2:**
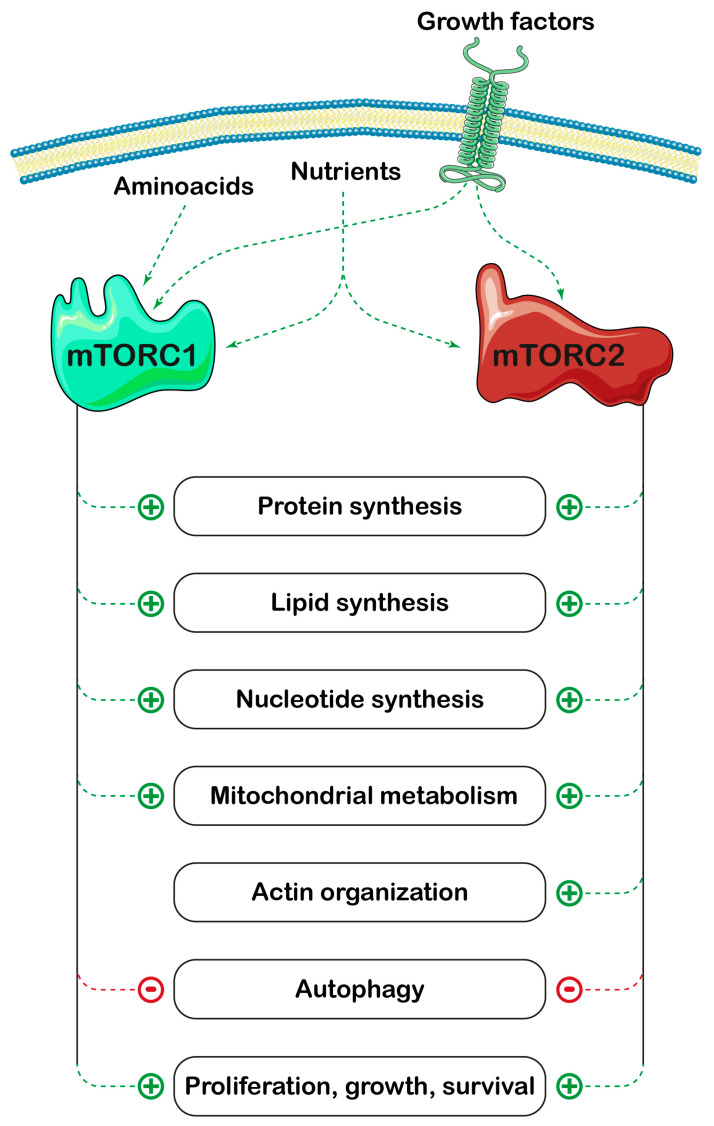
Effects of mTOR activation. mTOR promotes anabolic processes and inhibits autophagy, leading to proliferation, growth and survival. Parts of the figure were drawn by using pictures from Servier Medical Art. Servier Medical Art is licensed under CC BY 4.0, (To view the full details of this license, visit https://creativecommons.org/licenses/by/4.0/ last accessed on 9 May 2024).

Purine nucleotides are building blocks for nucleic acids, but also precursors of cofactors, central signaling compounds and essential molecules for bioenergetic processes. Their concentration depends on the balance between synthesis and degradation pathways (Figure 1). The synthesis of purine nucleotides proceeds through two major metabolic routes: the “de novo” and the salvage pathways. The “de novo” synthesis pathway is composed of six enzymes, which catalyze the ten steps of converting 5-phosphoribosyl-1-pyrophosphate (PRPP) into inosine-5′-monophosphate (IMP) (Figure 1). Besides PRPP, a precursor of the ribose-phosphate moiety of nucleotides, formed from ribose-5-phosphate (Rib-5-P) and ATP, other precursors are required for the “de novo” synthesis of the purine ring. These are amino acids (glutamine, aspartate and glycine), CO_2_, N^10^-formyl tetrahydrofolate (formyl THF) and ATP. From IMP, which is the final product of the purine “de novo” synthesis, both AMP and GMP are generated, which in turn can be converted back to IMP (Figure 1), thus constituting two interconverting pathways, the AMP and GMP cycles. The salvage of the preformed purine ring, which is less ATP-demanding as compared to the “de novo” synthesis, requires PRPP (for the salvage of adenine and hypoxanthine/guanine) or ATP (for adenosine salvage) (Figure 1). Purine nucleotides are subjected to catabolic processes, which start from the hydrolytic removal of phosphate, catalyzed by cytosolic 5′-nucleotidases, with the formation of the corresponding nucleosides. Adenosine is firstly deaminated to inosine by adenosine deaminase, and guanosine and inosine are phosphorolytically cleaved by purine nucleoside phosphorylase into the nucleobases, which are eventually converted to uric acid, the final product of purine catabolism in humans and great apes [1] (Figure 1). Both synthesis and degradation of purines are entirely cytosolic processes. Under purine-depleted growth conditions, the “de novo” purine enzymes assemble to form purinosomes [2], which cluster near the mitochondria [3], and enhance the metabolic flux through the pathway [4]. It is assumed that in normal physiological conditions most of the purine pool is generated through the salvage pathways, while, if cellular demands for nucleotide synthesis increase, the “de novo” biosynthetic pathway is upregulated [5,6]. Tumor cells reprogram their metabolic pathways to meet the increased requirement for nucleotides and other molecules necessary for their growth and proliferation [7,8,9]. In this regard, the “de novo” synthesis of nucleotides is enhanced in cancer cells [8], but also, salvage and interconverting enzymes appear to play a crucial role in both tumors and brain function [10,11]. Therefore, it is not surprising that specific enzymes of purine metabolism are targets of drugs as antineoplastic agents [12], and a better knowledge of the mechanisms underlying their regulation would be of great help in finding new therapeutic approaches. A key molecular determinant for “de novo” nucleotide synthesis has been identified in the mammalian target of the rapamycin (mTOR) signaling pathway [8,9]. mTOR is part of two distinct complexes, named mTOR complex 1 (mTORC1) and mTOR complex 2 (mTORC2). Overall, mTOR promotes anabolic processes, is a major regulator of cell growth and division and is frequently activated in cancer [13,14] (Figure 2). The mTOR pathway may exert short-term and long-term regulation, affecting growth and metabolism directly by phosphorylating target enzymes or indirectly by downstream signaling effectors [15]. Among the numerous effects exerted by mTOR, noteworthy is its empowerment of the “de novo” synthesis of nucleotides [8]. In this review, we emphasize the importance of mTOR in supporting the formation of purinosomes, and its stimulation of the “de novo” purine synthesis by increasing the availability of necessary precursors, such as one-carbon formyl group, bicarbonate and PRPP. Special attention will be given to the relationship between purine and mitochondrial metabolism, and to the bidirectional relationship between mTOR signaling and purine synthesis pathways.

## 2. Purine “De Novo” Synthesis and Mitochondria

Enzymes involved in purine “de novo” synthesis are cytosolic proteins. The flux through the pathway is regulated by enzyme expression that mainly depends on the cell cycle [16], by covalent modification [17] and also by enzyme aggregation and localization on microtubules and mitochondria forming purinosomes [2]. Therefore, mitochondria offer the purine “de novo” pathway a solid support for protein-protein aggregation that increases the flux through the pathway, both increasing the substrate concentration in the close vicinity of the enzymes and protecting unstable intermediates. In addition, the high concentration of purine near the mitochondria increases their flux through the mitochondrial membranes and possibly the rate of nucleic acid synthesis during mitochondrial proliferation. The rate of purine synthesis is dependent also on the precursor concentration, particularly glutamine, aspartate, glycine, PRPP and formyl THF.

### 2.1. Purinosome and Mitochondria

Clustering of the enzymes involved in the “de novo” purine synthesis was observed for the first time in 2008 when it was demonstrated that three enzymes in the pathway, namely PRPP amidotransferase (PPAT), glycinamide ribonucleotide transformylase (GART) and formyl glycinamide synthetase (FGAMS), interact forming the core of purinosome [2]. Further studies demonstrated that bifunctional carboxyaminoimidazole ribonucleotide synthetase/succinyl aminoimidazole carboxamide ribonucleotide synthetase (PAICS), adenylosuccinate lyase (ADSL) and 5-aminoimidazole-4-carboxamide ribonucleotide (AICAR) formyltransferase (AICAR-TF)/IMP cyclohydrolase (IMPCH), defined from now on as ATIC, interact together and with the core of purinosome [18]. Finally, IMP dehydrogenase (IMPDH) and adenylosuccinate synthetase (ADSS) have been demonstrated to be part of the purinosome complex, as well as the heat shock protein 90 (HSP90) and casein kinase II (CK2), which interact peripherally influencing the formation and the stabilization of the complex [19]. The purinosome clusters on mitochondria and on microtubules and is directed by microtubule transport proteins toward the mitochondria where it accumulates in the intersection with microtubules [20]. The purinosome formation is regulated by several physiological conditions, such as lack of preformed purine in the growth media of cells [2], mitochondrial dysfunction [3] and hypoxia [21]. It has been recently demonstrated that melanoma cells depend upon purinosome formation to meet the purine requirements to sustain their growth [22]. Applying several technical approaches, the involvement of some signaling pathways and protein kinases have been proven to be involved in the regulation of purinosome formation and localization on mitochondria. Purinergic receptors, such as A2A adenosine receptors and also other signals activating the G-protein-coupled receptor (GPCR), were demonstrated to positively influence purinosome formation [23]. Furthermore, CK2 can phosphorylate the three enzymes forming the core of the purinosome, preventing their clustering [24]. More recently, it was demonstrated that extracellular signal-regulated kinase 2 (ERK2) phosphorylates FGAMS, increasing its activity in cells stimulated by growth factors [25].

mTOR has been found to regulate the flux of purine “de novo” synthesis by acting on enzyme expression, on covalent modification and also on the availability of the substrates [15]. 

French et al. [3] demonstrated that mTOR regulates the binding of purinosomes on mitochondria. Furthermore, the increase in purinosomes present on the mitochondria of cells with mitochondrial dysfunction or stimulated by agonists of GPCR is also dependent on mTOR [3]. Therefore, mTOR increases the mRNA translation of the rate limiting enzymes of purine nucleotides, increasing the “de novo” synthesis [26] and also regulates in a posttranslational way the aggregation of purine synthesis enzymes on mitochondria. The mTOR regulation occurs following not only proliferation stimuli but also signals of mitochondrial dysfunction or oxygen scarcity. The purinosome also contains chaperones, such as HSP70 and HSP90. Several studies indicate that the function of these proteins is to maintain the correct folding for aggregation of the enzymes, forming the core of the purinosome [27]. It was recently demonstrated that HSP90 is essential for the correct assembly of the purinosome in normoxic cells growing in purine-free media [28], while HSP70 appears to be important, particularly for the formation of purinosome induced by hypoxia but not in normoxia [21]. The transcription of HSP is mediated by HSF1, a transcription factor activated by stressful conditions. The regulation of the central function of HSF1 relies on several posttranslational modifications, including a serine phosphorylation mediated by mTORC1. In fact, the mutation of that serine residue completely prevents the increase in HSP synthesis induced by stress [29]. While the mechanism leading to purinosome formation during hypoxia in HeLa cells is not clear, the aggregation of the “de novo” synthesis enzymes on mitochondria is not accompanied by an increase in flux through the pathway, probably because, in hypoxic conditions, the one-carbon metabolism is down regulated in mitochondria, limiting the rate of the “de novo” synthesis (see next section) [21]. 

The clustering of enzymes of purine “de novo” synthesis on mitochondria to increase the flux of the pathway was supposed to be linked to the high concentration of ATP around the organelles, necessary to provide the pathway with a high concentration of high-energy compounds. Nevertheless, it was demonstrated that the formation of purinosome is independent of ATP concentration [3].

The mTOR enzyme has been also indicated as the regulator of pyrimidine synthesis [30]. The mTORC1 complex, which is usually activated by growth factors in the presence of high concentration of nutrients, phosphorylates ribosomal protein S6 kinase beta 1 (S6K1), a protein kinase that covalently modifies CAD (carbamoyl-phosphate synthetase-2/aspartate transcarbamoylase/dihydroorotase), the trifunctional enzyme, which catalyzes the first three steps of pyrimidine “de novo” synthesis, thus increasing the rate of the pathway [30]. It is worth remembering that the enzyme dihydroorotate dehydrogenase, which catalyzes the fourth step of the pathway, is a mitochondrial enzyme and part of the oxidative phosphorylation machinery since it transfers electrons from its substrate to coenzyme Q [31].

### 2.2. Mitochondria and One-Carbon Metabolism

As stated above, mitochondria are essential not only for ADP phosphorylation but also for sustaining a high rate of nucleotide synthesis. Warburg’s theory on the origins of cancer postulates that tumor cells have defects in mitochondrial oxidative phosphorylation and, therefore, rely on high levels of aerobic glycolysis as the major source of ATP to fuel cellular proliferation [32]. Nevertheless, the function of these organelles is essential for proliferation also in tumor cells. In fact, it has been demonstrated that, in the absence of mitochondrial DNA, tumorogenesis is impossible [33]. Mitochondrial genome acquisition restores respiratory function and tumorigenic potential in cancer cells without mitochondrial DNA [7]. 

**Figure 3:**
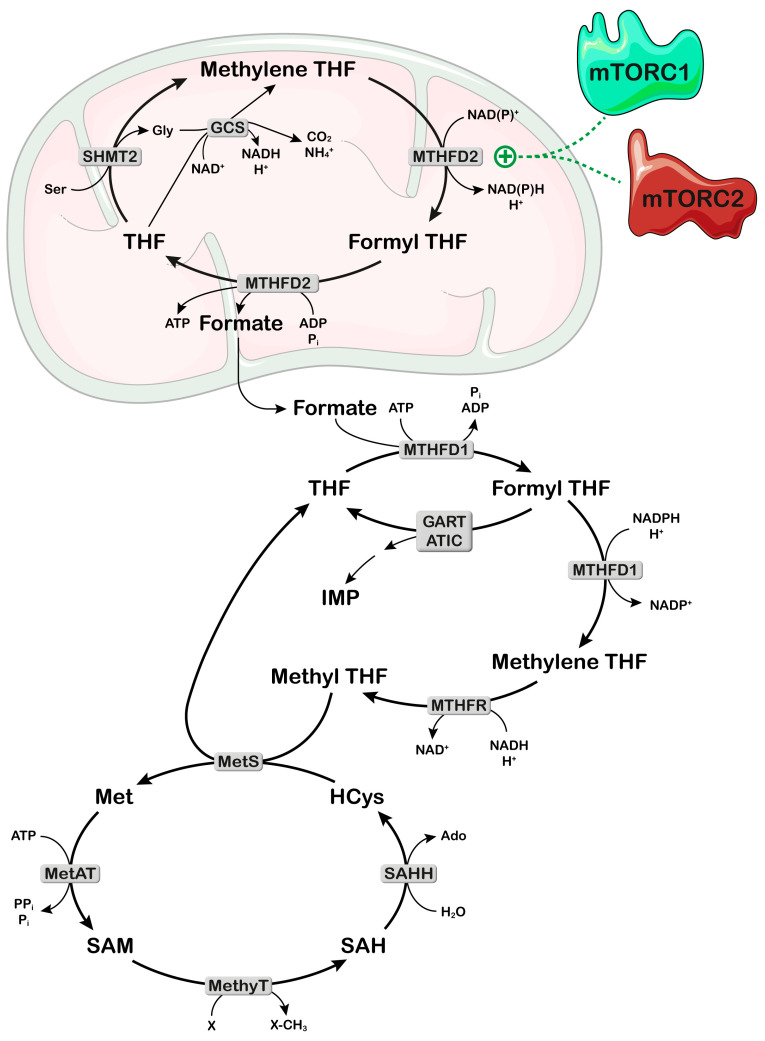
mTOR affects one-carbon metabolism and purine “de novo” synthesis. Inside the mitochondria the methylene group of Ser (serine) is transferred to THF (tetrahydrofolate) through SHMT2 (serine hydroxymethyltransferase-2), forming methylene-THF. Methylene-THF, which can be obtained also by GCS (glycine cleavage system), is oxidized to formyl THF and the formyl group is released in a reaction catalyzed by MTHFD2 (methylene THF dehydrogenase-2), which is activated by mTOR. The formyl group leaves the mitochondria and recombines with cytosolic THF. Formyl THF can be utilized as a one-carbon donor in the “de novo” synthesis of purine or be reduced to methylene THF by the enzyme MTHFD1. A further reduction of methylene THF, catalyzed by MTHFR (MTHF reductase), generates methyl-THF, essential for the conversion of HCys (homocysteine) into Met (methionine) catalyzed by MetS (methionine synthase). MetAT (methionine adenosyl transferase) converts Met in SAM (S-adenosyl methionine), which functions as a methyl donor in methylation reaction, catalyzed by methylT (methyl transferase). GART: glycinamide ribonucleotide transformylase; ATIC: 5-aminoimidazole-4-carboxamide ribonucleotide formyltransferase/IMP cyclohydrolase. Parts of the figure were drawn by using pictures from Servier Medical Art. Servier Medical Art is licensed under CC BY 4.0, (To view full details of this license, visit https://creativecommons.org/licenses/by/4.0/ last accessed on 9 May 2024).

One-carbon metabolism is essential for purine “de novo” synthesis since it contributes the formyl group from which two carbon units of the purine molecule derive (Figure 1). The carbon unit is essentially obtained from serine, which donates its methylene group and is converted into glycine (Figure 3) [34]. The carbon unit can also be obtained by the glycine cleavage system, but it has been demonstrated that, during high-rate purine synthesis, it is synthesized mainly from serine [35]. The compound involved in the transport of the one-carbon unit is THF. The great importance of folates for cell proliferation has been known for many years and is the basis of numerous chemotherapy compounds, such as methotrexate and pemetrexed [36]. The enzymes involved in one-carbon metabolism are present both in the cytosol and inside the mitochondria [34]. However, the one-carbon unit, essential for purine synthesis, originates mainly from inside the mitochondria (Figure 3) [35]. Serine transfers its methylene group to THF, forming glycine through the action of mitochondrial serine hydroxymethyltransferase-2 (SHMT2). Methylene THF is oxidized by mitochondrial methylene THF dehydrogenase-2 (MTHFD2) to formyl THF, which releases its formyl group generating ATP. The monocarbon unit can freely cross the mitochondrial membrane and enter the cytosol, where it recombines with cytosolic THF with ATP consumption (Figure 3). The formyl THF participates in purine “de novo” synthesis or is reduced back to methylene THF by cytosolic MTHFD1. Methylene THF can be further reduced to methyl THF in an irreversible reaction catalyzed by methylene THF reductase (MTHFR). The methyl group generated in this cycle can be utilized for the conversion of homocysteine into methionine in the presence of vitamin B12. This reaction is essential for the synthesis of S-adenosyl methionine involved in the methylation of several targets [35] (Figure 3).

Lewis et al. [37] demonstrated that in mitochondria the THF metabolism proceeds toward the formation of both NADPH and NADH, while in the cytosol the formyl THF can be reduced to methylene THF at the expense of NADPH generated by the pentose phosphate pathway (PPP). This THF cycle, essential for purine synthesis and DNA methylation, is regulated by both mTORC1 and mTORC2. In fact, both complexes activate the activation transcription factor 4 (ATF4), which in turn increases the translation of MTHFD2 (Figure 3) [38,39]. The same transcription factor also upregulates some enzymes involved in serine synthesis [38]. The non-essential amino acid serine is important for several processes besides nucleotide synthesis, which are basilar for the growth and survival of proliferating cells, such as protein, amino acid, glutathione synthesis, methylation reactions and generation of NADPH for antioxidant defense [40]. Indeed, many cancer cells are highly dependent on exogenous serine or rely on an upregulation of the enzymes involved in serine synthesis [40].

In conclusion, mTOR is involved in the regulation of purine “de novo” synthesis in many ways. It increases the transcription of the protein involved in the pathway, including HSP essential for purinosome formation and stabilization [15,29]. It regulates the availability of nucleotide precursors, such as formyl THF and, as described in the following section, PRPP. The mTOR enzyme also regulates purinosome formation in many different stressful conditions when the ability to rapidly synthesize nucleotides may help to save cell life [3]. In fact, in the case of mitochondrial dysfunction and hypoxia, the possibility of sustaining mitochondrial proliferation may help to improve metabolic performances. The relationship between mTOR activation and purine synthesis is further demonstrated by the regulation of colon epithelium regeneration operated by the RNAse activity of Regnase-1 that controls purine synthesis and proliferation regulating mTOR [41].

## 3. mTOR Signaling Controls Availability of Both Bicarbonate and PRPP for Nucleotide Synthesis

**Figure 4:**
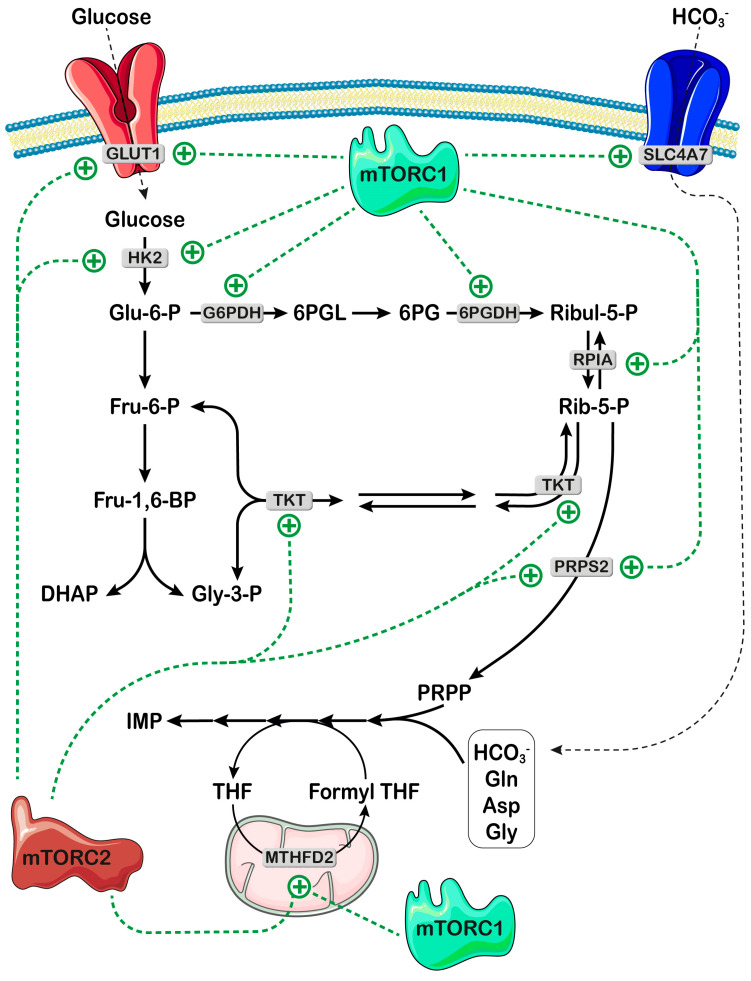
mTOR signaling controls the availability of both bicarbonate and PRPP for nucleotide synthesis. mTORC1 favors the entry and entrapment of glucose by increasing the expression of the glucose transporter (GLUT1) and HK2 (hexokinase 2). mTORC1 also activates the pentose phosphate pathway by enhancing the transcription of genes encoding for both G6PDH (glucose-6-phosphate dehydrogenase) and 6PGDH (6-phosphogluconate dehydrogenase). The conversion of Ribul-5-P (ribulose-5-phosphate) in PRPP (5-phosphoribosyl-1-pyrophosphate) is favored by the activation of RPIA (ribose-5-phosphate isomerase A) and PRPS2 (PRPP synthetase 2). mTORC2 increases both glucose transporter GLUT1 and HK2 expression and activates TKT (transketolase) and PRPS2. mTORC1 controls the availability of bicarbonate by enhancing the expression of the sodium and bicarbonate cotransporter SLC4A7. Bicarbonate is then used for the “de novo” synthesis of IMP. Both mTOR complexes promote the formation of formyl THF, through activation of mitochondrial MTHFD2 (methylene THF dehydrogenase-2) (see also Figure 3). Glu-6-P: glucose-6-phosphate; Fru-6-P. fructose-6-phosphate; Fru-1,6-BP: Fructose-1,6-bisphosphate; DHAP: dihydroxyacetone phosphate; Gly-3-P: glyceraldehyde-3-phosphate; Gln: glutamine; Asp: aspartate; Gly: glycine; THF: tetrahydrofolate. Parts of the figure were drawn by using pictures from Servier Medical Art. Servier Medical Art is licensed under CC BY 4.0, (To view full details of this license, visit https://creativecommons.org/licenses/by/4.0/ last accessed on 9 May 2024).

Ali et al. [42] demonstrated that bicarbonate is a limiting substrate for the “de novo” synthesis of both purine and pyrimidine nucleotides in proliferating cells. To promote the entry of bicarbonate in proliferating cells, mTORC1, in response to growth factors, stimulates the mRNA translation of sodium and bicarbonate cotransporter SLC4A7 through S6K-dependent phosphorylation of the eukaryotic translation initiation factor 4B (eIF4B), thus increasing the amount of SLC4A7 protein [42] (Figure 4). Among ten bicarbonate transporters of the SLC4A family, only SLC4A7 was stimulated by mTORC1 in response to growth signals [42]. Therefore, SLC4A7 appears to play a key role in favoring nucleotide synthesis in tumors and tumor growth. Indeed, SLC4A7 loss or knockdown reduces proliferation in several cancer cell lines and sensitizes tumors to mTOR inhibition [42], thus encouraging the development of therapeutic strategies targeting SLC4A7 for the treatment of cancer.

PRPP synthetase (PRPS) catalyzes the formation of PRPP from ATP and Rib-5-P (Figure 1) and is composed of isoforms 1 and 2, sharing 95% homology [43]. Although PRPS is not part of the purinosome, its central importance in the “de novo” purine synthesis pathway is highlighted not only by its complex regulation (glycosylation [44], phosphorylation by AMP-activated protein kinase (AMPK) [45], feedback purine nucleotide inhibition [46]), but also by its role as a supplier of PRPP, one of the two substrates of PPAT, the first and rate-limiting enzyme of the “de novo” purine pathway (Figure 1) [46]. It must be emphasized that the PRPP is also the obligated substrate for the salvage of purine and pyrimidine bases into their respective mononucleotides and for the synthesis of pyridine nucleotides [10,47]. Therefore, the concentration of PRPP and the rate of purine synthesis are closely interconnected [48]. The availability of PRPP appears to influence purinosome formation; indeed, in fibroblasts of Lesch-Nyhan patients, where the deficiency of hypoxanthine-guanine phosphoribosyl transferase causes an accumulation of PRPP [49], the purinosome frequency has been reported to be elevated [50]. Rib-5-P, the necessary precursor of PRPP, is generated from the oxidative and non-oxidative branches of the PPP. The mTOR enzyme appears to play a central role in accelerating both glycolysis and PPP, thus increasing the availability of both NADPH and Rib-5-P, and its deregulation meets the increased demand for lipids and nucleotides of cancer cells. Indeed, through hypoxia-inducible factor 1α (HIF1α) and Myc, mTORC1 and mTORC2 increase both glucose transporter GLUT1 and hexokinase 2 expressions [15,51], thus favoring entry and entrapment of glucose inside the cell. In addition, mTORC1 has been reported to enhance the transcription of genes encoding for glucose-6-phosphate dehydrogenase and 6-phosphogluconate dehydrogenase [51] and both the transcription [51] and the translation [52] of ribose-5-phosphate isomerase A. Furthermore, mTORC2 controls the activity of transketolase through Akt, which phosphorylates and activates the enzyme [53]. Therefore, mTOR contributes to increasing the concentration of Rib-5-P by upregulating the enzymes involved in its formation (Figure 4). Finally, both mTOR complexes appear to control the activity of PRPS2 through Myc-mediated translational upregulation of the enzyme [54]. In fact, *PRPS2*, unlike *PRPS1*, harbors a pyrimidine-rich translational element located in its 5′UTR, which enables translational regulation by Myc to increase nucleotide synthesis [54]. In addition, the isoform 2 of PRPS is more resistant to nucleotide feedback inhibition [43,55], thus contributing to the deregulation of the “de novo” synthesis pathway in Myc-overexpressing cancer cells. Nucleotides are, in turn, required to synthesize ribosomes, necessary for increased protein synthesis rates. In this regard, PRPS2 can couple protein and nucleotide biosynthesis, and, as suggested by Cunningham et al. [54], drugs capable of specific inhibition of this isoform of the enzyme may offer a therapeutic window for Myc-overexpressing cancer cells.

## 4. Effect of Purine Metabolizing Enzymes on the mTOR Signaling Pathways

**Figure 5:**
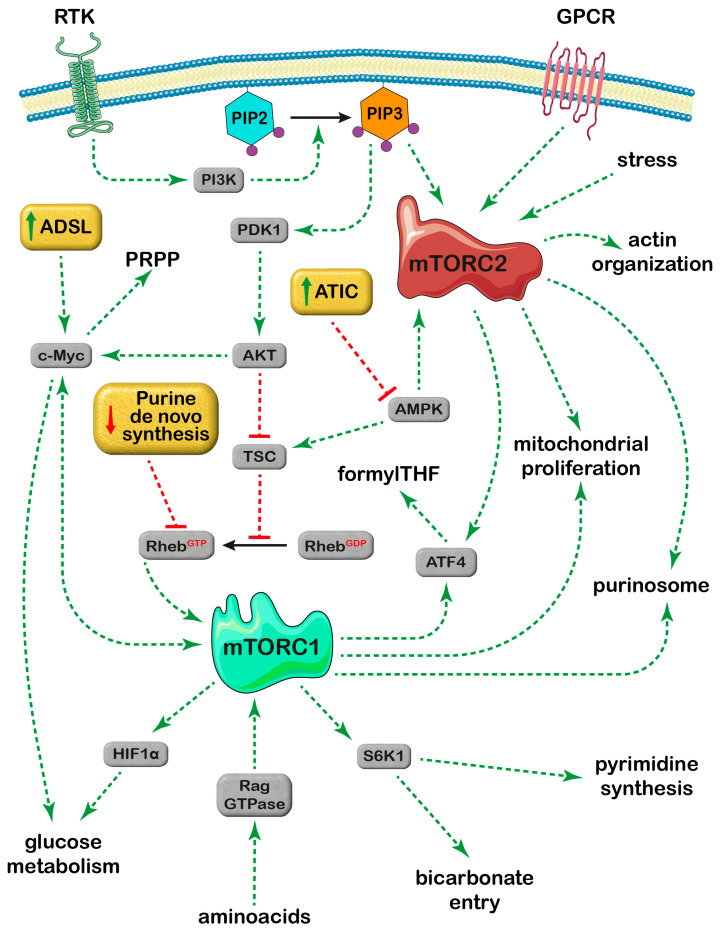
Pathways of mTORC1 and mTORC2 regulation. The figure includes only the signaling pathways described in the text. RTK (tyrosine kinase receptor) activates PI3K (phosphatidylinositol-3-kinase), which catalyzes the phosphorylation of PIP2 (phosphatidylinositol-4,5-bisphosphate) into PIP3 (phosphatidylinositol-3,4,5-trisphosphate). PIP3 activates mTORC2 and PDK1 (phosphoinositide-dependent kinase-1). PDK1 phosphorylates Akt, which in turn activates c-Myc and inhibits TSC (tuberous sclerosis complex). TSC inactivates Rheb (Ras homolog enriched in the brain) and inhibits Rheb-mediated activation of mTORC1. AMPK (AMP-activated protein kinase) activates both TSC and mTORC2. Both mTOR complexes activate ATF4 (activating transcription factor 4). GPCR: G-protein-coupled receptor; S6K1: ribosomal protein S6 kinase beta 1; Rag: Ras-related GTP binding protein; HIF1α: hypoxia-inducible factor 1α; ADSL: adenylosuccinate lyase; ATIC: 5-aminoimidazole-4-carboxamide ribonucleotide formyltransferase/IMP cyclohydrolase. Red: inhibition. Green: activation. Parts of the figure were drawn by using pictures from Servier Medical Art. Servier Medical Art is licensed under CC BY 4.0, (To view full details of this license, visit https://creativecommons.org/licenses/by/4.0/ last accessed on 9 May 2024).

The interaction of the mTOR signaling and purine nucleotide pathways is not unidirectional. Indeed, purine nucleotide depletion, obtained by inhibition of enzymes of the “de novo” synthesis, has been reported to suppress mTORC1 activity [56,57]. The inhibition, which is purine-, but not pyrimidine-dependent, is rescued by the addition of purine nucleobases and appears to be dependent on the dramatic reduction of GTP-bound Ras homolog enriched in brain (RhebGTP), the functionally active form of Rheb [57] and/or on the GTPase-activating protein of tuberous sclerosis complex (TSC) and its regulation of Rheb [56] (Figure 5). In this light, it would be interesting to investigate whether the anti-neoplastic effect of inhibitors of the purine synthesis (such as methotrexate and 6-mercaptopurine) is due, at least in part, to mTORC1 activity suppression. The interplay between nucleotide availability and mTOR activity could be exploited for a more targeted cancer therapy. As an example, Valvezan et al. [58,59] reported that IMP dehydrogenase (IMPDH) inhibition selectively kills TSC-deficient tumor cells, which are not able to inhibit Rheb-mediated activation of mTORC1 (Figure 5) and are therefore characterized by a strong mTORC1 stimulation [60]. The rationale for the use of the IMPDH inhibitor, mizoribine, in TSC-deficient tumors with active mTORC1, relies on the depletion of guanylate nucleotides (see Figure 1), which are particularly required to meet the increased demand caused by mTORC1-driven rRNA synthesis. This renders nucleotides limiting for DNA replication, thus causing replication stress, DNA damage and apoptotic cell death [58,59]. It must be emphasized that the selective cytotoxic action of IMPDH inhibitors is dependent on operative mTORC1 signaling; therefore, they should not be used in combination with rapalogs, which are approved drugs to treat TSC-deficient tumors [61]. However, the rapalog effect appears to be mainly cytostatic, rather than cytotoxic, and tumors regrow when the treatment is suspended [62].

As reported above, an inhibition of purine synthesis appears to be associated with a suppression of the mTOR signaling pathways [56,57]. However, in several types of tumors, activation of mTOR has been reported to be determined by the upregulation of purine “de novo” synthesis enzymes, namely ADSL [63] and ATIC [64,65]. As outlined in the following sections, deciphering the pathways connecting these purine enzyme activities and mTOR signaling may offer new therapeutic targets in these tumor types.

### 4.1. ADSL and mTOR

ADSL participates in two steps of purine biosynthesis. The enzyme catalyzes the formation of AICAR from succinyl-AICAR (SAICAR) in the “de novo” pathway, and also the conversion of succinyl-AMP (S-AMP) into AMP, thus participating in the “AMP cycle” (Figure 1). ADSL deficiency is a rare genetic disorder, causing severe neurological symptoms, the etiology of which is still under investigation [66]. Conversely, the expression of ADSL has been found to significantly increase in several tumors [63,67,68,69] and the prevalence of ADSL-rs3788579 polymorphism has been recently reported in female cancer patients in North-West Iran [70]. In colorectal carcinoma (CRC) tumors [63] and in triple negative breast cancer (TNBC) [67], ADSL has been reported as an oncogenic driver acting through the mTORC1-Myc signaling pathway. In TNBC, where ADSL shows the highest expression as compared to other breast cancers [67], the enzyme has been demonstrated to be the substrate of EglN2 hydroxylase [67], and this hydroxylation is important for maintaining enzymatic activity. This hydroxylation is a driver of TNBC proliferation and invasion, and a positive correlation has been found between ADSL and c-Myc expression [67]. In fact, ADSL negatively controls the long non-coding RNA *MIR22HG*, which, in turn, negatively regulates the expression of c-Myc. Among the products of the reaction catalyzed by ADSL (AMP, fumarate, AICAR) (see Figure 1), Zurlo et al. [67] identified adenosine, formed from AMP dephosphorylation, as responsible for the repression of the long non-coding RNA *MIR22HG* expression in TNBC. The upregulation of c-Myc, observed in TNBC, was accompanied also by an activation of mTORC1 [67]. Indeed, at least in hepatocarcinogenesis, a functional interaction exists between mTOR and c-Myc cascades [71]. 

In CRC samples, Taha-Mehlitz et al. [63] demonstrated that ADSL hyperexpression induced dysregulation of the Krebs cycle (even though they excluded the formation of fumarate as the triggering event) and mitochondrial dysfunction, with the accumulation of ROS and activation of mTORC1 and c-Myc. Their data cannot discriminate whether mitochondrial dysfunction with subsequent ROS production causes or is caused by activation of the mTORC1-cMyc pathway and whether mTORC1 activation increases c-Myc or vice versa.

Recently, Yin et al. [72] reported that ADSL, which is upregulated in glioma stem cells (GSCs), is responsible for the formation of fumarate, necessary for the succination of phosphatase and tensin homolog (PTEN), a posttranslational modification, which diminished its inhibitory effect on the phosphatidylinosi-tol-3-kinase (PI3K)/Akt pathway. PTEN is, in fact, a tumor suppressor, which dephosphorylates phosphatidylinositol-3,4,5-trisphosphate [73], thus inhibiting PI3K signaling (see Figure 5). It is conceivable that, through this mechanism, ADSL may contribute to mTOR activation in GSCs, although the authors did not take this aspect into consideration.

Even though more in-depth studies are needed to unravel the molecular pathways through which ADSL promotes tumor growth, it is undoubtedly true that their knowledge may contribute to the design of novel therapeutic strategies.

### 4.2. ATIC and mTOR

ATIC catalyzes a two-step reaction of the “de novo” purine synthesis, in which AICAR-TF (C-terminal domain) catalyzes the formation of formyl-AICAR (FAICAR) and IMPCH (N-terminal domain) cyclizes FAICAR to IMP (Figure 1). ATIC is therefore responsible for the control of the levels of AICAR, which is an AMP mimetic known to activate AMPK [74]. Therefore, ATIC belongs to the family of adenylate metabolizing enzymes that regulate the levels of AMP (or AICAR) and hence, the activity of AMPK [75], the master regulator of cellular energy homeostasis [76]. A deficiency of ATIC was reported in a patient presenting with devastating neurological symptoms [77], while a hyperexpression of ATIC has been found in several tumoral tissues, as compared to normal tissues [64,65,78,79]. An upregulation of ATIC expression was reported in samples of human hepatocellular carcinoma (HCC), and high levels of ATIC appeared to be a good marker of poor prognosis in HCC patients [64]. To address this issue, Li et al. [64] knocked down ATIC expression in HCC cell lines and found a correlation with an increased intracellular level of AICAR, which in turn, activated AMPK. Among the plethora of targets of activated AMPK, the authors identified, in the AMPK-mediated inhibition of the mTORC1-S6K1 signaling pathway, the pathway responsible for the decreased cell growth and proliferation observed in ATIC-knockdown HCC cells (Figure 5) [64]. Therefore, the authors inferred that ATIC, the activity of which is increased in HCC, may drive tumor progression by regulating the AMPK-mTOR-S6K1 signaling [64]. ATIC was also found to inhibit autophagy and to promote liver cancer progression through the Akt/Forkhead box subgroup O3 (FOXO3) pathway [79]; however, the possible involvement of mTOR in the process was not investigated.

A higher expression of ATIC was found in lung adenocarcinoma (LUAD) tissues and a positive association with Myc expression was reported [65]. By using LUAD cell lines, Niu et al. [65] demonstrated that ATIC hyperexpression increased cell growth, migration and invasion, while ATIC silencing led to the suppression of cell growth, migration and invasion. The authors also verified that the increased expression of Myc was related to mTOR activation [65]. Although not specifically investigated, it is conceivable that the level of AICAR may regulate the activity of AMPK and hence its downstream effector mTOR.

In light of the reported studies, ATIC may represent a novel diagnostic marker and a potential molecular target for HCC and LUAD therapies.

## 5. Discussion

A high availability of nucleotide precursors is essential for tumor growth, and usually, nucleotide synthesis is a rate limiting factor for proliferation [80]. The nucleotide synthesis requires chemical energy, both carbon and nitrogen precursors and multiple inputs from different pathways and organelles, particularly mitochondria [81]. For these reasons, both the nucleotide synthesis pathway and the related mitochondrial pathways have been targeted by chemotherapy for decades. The typical approach is the inhibition of the synthesis pathways using purine analogs that function as competitive inhibitors, which negatively affect DNA integrity and replication, eventually causing apoptosis [82]. It is conceivable that these analogs, which inhibit the “de novo” synthesis, also inhibit mTOR [56,57], further decreasing tumor proliferation. These compounds represent now a significant percentage of the available treatments for cancer [81]. 

The regulation of the rate of nucleotide synthesis is essential for proliferating cells and particularly for tumor cells that require their metabolism to be altered by hyperexpression of oncogenes and hypoexpression of tumor suppressor genes, to support the rapid transformation of nutrients into cellular biomass [83]. In tumors, a number of genes encoding for proteins activated by mTOR signaling pathways are listed as oncogenes, while proteins involved in the down regulation or control of mTOR activity are known as tumor suppressors [84].

The mTORC1 complex is a sensor of growth factors, nutrients, particularly aminoacids, and oxygen availability, and on the basis of these inputs, its activation starts proliferation by increasing nucleotide and protein synthesis [85]. The mTOR pathway and therefore, nucleotide synthesis, can be activated also by stressful conditions [3,21], thus activating mechanisms necessary for survival. The mTORC2 signaling pathway can be triggered by both anabolic and catabolic signals. In fact, mTORC2 can be activated by PI3K signals [86] but also by AMPK during glucose starvation [87]. Further studies are necessary to address how mTORC2 can be distinctly regulated by such opposite inputs. The molecular mechanisms underlying the response of mTORC2 to stressful conditions are poorly understood, but once activated, mTORC2 initiates the Akt signaling pathway, which can mediate survival in hypoxic conditions or in case of mitochondrial dysfunction activating a plethora of signals, including the transcription factor c-Myc [88] (Figure 5). Also, mTORC1 may activate c-Myc, which in turn favors both glycolytic gene expression and mitochondrial respiration [71], thus promoting survival.

To distinguish which mTOR complex is involved in the regulation of a particular cell mechanism, researchers rely on the use of rapamycin, which specifically inhibits mTORC1, while, so far, no specific inhibitors for mTORC2 are available [89]. However, while short incubations with rapamycin inhibit mTORC1, prolonged incubations indirectly inhibit also mTORC2 since rapamycin can target newly synthesized mTOR, thus preventing the assembly of novel mTORC2 [90]. Further complexity is added by the ability of mTORC2 to bind to different membranes [89]. This localization enhances phosphorylation and activation of the substrates in close vicinity, starting different signaling pathways, which depend on mTORC2 compartmentalization [89]. Finally, a strict cross-talk exists between mTORC2 and mTORC1, which can be realized through different pathways, including Akt activation by mTORC2, with a subsequent mTORC1 activation [89] (Figure 5). The formation of purinosomes is dependent on mTOR and is inhibited by rapamycin, indicating a possible involvement of mTORC1 but is also regulated through GPCR, known to modulate mTORC2 activity [3,89]. Therefore, both mTOR complexes appear to play a relevant role in the formation of purinosomes. In conclusion, while more work is necessary to understand the molecular mechanisms underlying the modulation of purine “de novo” synthesis, several researchers agree that dysregulation of purine “de novo” synthesis, as well as dysregulation of mTOR functions, are at the basis of carcinogenesis [8,91,92].
